# Association between serum β_2_-microglobulin levels and the risk of all-cause and cardiovascular disease mortality in chinese patients undergoing maintenance hemodialysis

**DOI:** 10.1186/s12882-023-03191-5

**Published:** 2023-06-13

**Authors:** Yu-Xin Jin, Shuang Zhang, Jia Xiao, Zhi-Hong Wang, Cui Dong, Lian-Lian You, Ting-Ting Kuai, Yu Zhang, Shu-Xin Liu

**Affiliations:** 1 Graduate School, Dalian Medical University, Dalian, China; 2 Dalian Key Laboratory of Intelligent Blood Purifcation, Dalian Municipal Central Hospital affiliated with Dalian University of Technology, Dalian, China; 3 Department of Nephrology, Dalian Municipal Central Hospital affiliated with Dalian University of Technology, No.826, Xinan Road Dalian, 116033 Liaoning, P. R. China; 4 School of Clinical Medicine, Faculty of Medicine, Dalian University of Technology, Dalian, China

**Keywords:** β_2_-microglobulin, Cohort study, Hemodialysis, Mortality, Cardiovascular events

## Abstract

**Background:**

The association between serum β_2_-microglobulin (β_2_M) levels and the risk of all-cause and cardiovascular disease (CVD) mortality and the incidence of cardiovascular events (CVEs) in patients undergoing maintenance hemodialysis (MHD) is inconclusive. Furthermore, no study has been performed in China on the significance of serum β_2_M levels in MHD patients. Therefore, this study investigated the aforementioned association in MHD patients.

**Methods:**

In this prospective cohort study, 521 MHD patients were followed at Dalian Municipal Central Hospital affiliated with Dalian University of Technology from December 2019 to December 2021. The serum β_2_M levels were categorized into three tertiles, and the lowest tertile served as the reference group. Survival curves were calculated by the Kaplan–Meier method. Hazard ratios (HRs) and 95% confidence intervals (CIs) were calculated using Cox proportional hazard models. Sensitivity analysis was performed by excluding patients with CVD at baseline.

**Results:**

During the follow-up period of 21.4 ± 6.3 months, there were 106 all-cause deaths, of which 68 were caused by CVD. When excluding CVD patients at baseline, there were 66 incident CVEs. Kaplan–Meier analysis revealed that the risk of all-cause and CVD mortality in the highest tertile of serum β_2_M levels was significantly higher than that in the lowest tertile (*P* < 0.05), but not for the CVEs (*P* > 0.05). After adjusting for potential confounders, serum β_2_M levels were positively associated with the risk of all-cause (HR = 2.24, 95% CI = 1.21–4.17) and CVD (HR = 2.54, 95% CI = 1.19–5.43) mortality, and a linear trend was evident (*P* < 0.05). Besides, the results of sensitivity analysis were consistent with the main findings. However, we didn’t observed the significant association between serum β_2_M levels and CVEs (*P* > 0.05).

**Conclusion:**

The serum β_2_M level may be a significant predictor of the risk of all-cause and CVD mortality in MHD patients. Further studies are needed to confirm this finding.

## Introduction

End stage renal disease (ESRD) is defined by a glomerular filtration rate (GFR) of < 15 ml/min/1.73 m^2^ [1] and may be treated by kidney replacement therapy, which involves either dialysis or transplantation with supportive care [2]. The global incidence of ESRD was approximately 809,103 individuals in 2019, and approximately 85.1% of patients on dialysis received maintenance hemodialysis (MHD) [3]. However, patients undergoing MHD have a high risk of death. The United States Renal Data System showed that the mortality rate was 159.3 for MHD patients per 1,000 patient-years in 2019 [3]. Unfortunately, there is no national surveillance system for kidney diseases in China, and the annual mortality rate of MHD patients is 7.6–9% in Beijing, the capital city of China, where the level of medical care is well developed [4]. The mortality rates of other regions are expected to be higher. Cardiovascular disease (CVD) is the most common cause of death in MHD patients [5], and the risk of CVD mortality is 10–20 times higher in the MHD population than in the general population [6]. This is because MHD patients not only have classical risk factors for CVD, such as hypertension, diabetes mellitus, dyslipidemia, and hyperuricemia, but also have many non-classical chronic kidney disease (CKD)-specific risk factors for CVD, including anemia, volume overload, mineral bone disorders, inflammation, malnutrition, and activation of sympathetic nervous system and renin-angiotensin-aldosterone systems [7]. Furthermore, in recent years, increasing evidence suggests that uremic toxins (UTs) are non-classical CKD-specific risk factors [8–11].

Generally, UTs are substances that accumulate in the body following decreased kidney function [8, 12]. UTs are classified into three groups, namely, small water-soluble solutes, medium molecules, and protein-bound solutes [8, 9]. β_2_-Microglobulin (β_2_M) is a representative medium-molecule UT with a molecular weight of 11.729 kDa [13], which is produced by all cells expressing major histocompatibility class I [14, 15], and serum levels can be measured by latex immunoassay [16]. β_2_M is filtered by the glomerulus and degraded in the proximal tubules through a megalin-dependent pathway [17], and serum levels vary between 1 mg/L and 3 mg/L in healthy individuals [18, 19]. In dialysis patients, in whom the GFR is almost completely abolished, serum β_2_M levels can reach 20 mg/L to 50 mg/L or even higher [15]. Several studies have examined the association between serum β_2_M levels and clinical outcomes in MHD patients, but the results were inconclusive. The HEMO study showed that high serum β_2_M levels can significantly increase the risk of mortality [20]. By contrast, Kim et al. performed a retrospective cohort study in Korea and indicated that higher serum β_2_M levels were associated with lower mortality [21]. In the Chronic Renal Insufficiency Cohort (CRIC) Study, Foster et al. found that higher serum β_2_M levels was associated with increased incidence of cardiovascular events (CVEs) in CKD patients [22]. However, a cohort study in the Systolic Blood Pressure Intervention Trial (SPRINT) found that serum β_2_M levels were not associated with the incidence of CVEs [23].

To the best of our knowledge, in China, no studies have been performed to assess the role of serum β_2_M levels in predicting clinical outcomes in MHD patients. Thus, we conducted this prospective cohort study to explore the association between serum β_2_M levels and the risk of all-cause and CVD mortality and the incidence of CVEs in MHD patients.

## Materials and methods

### Study design and population

**Fig. 1:**
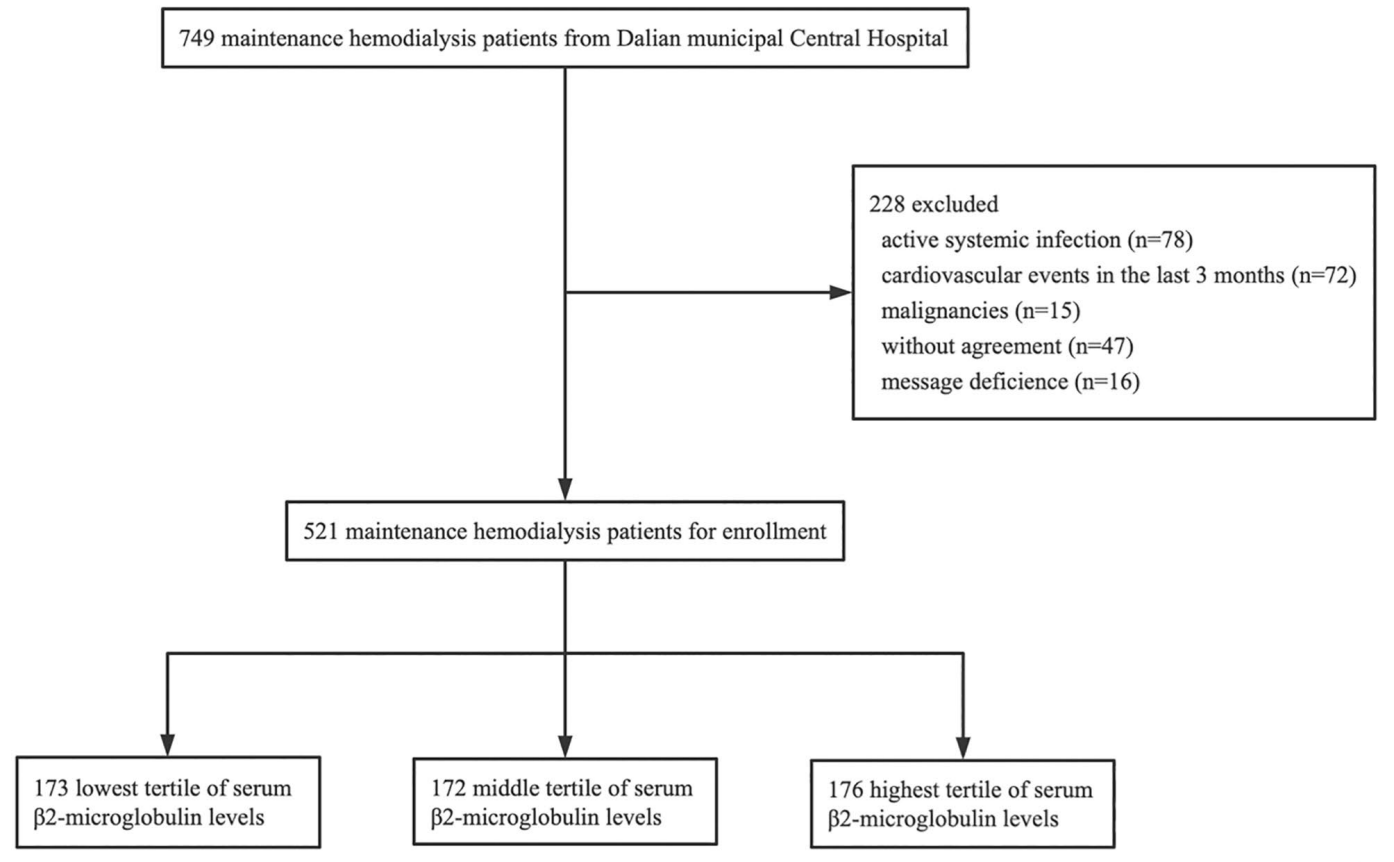
Flow chart indicates patient enrollment

This prospective cohort study enrolled 749 MHD patients from December 2019 from Dalian Municipal Central Hospital affiliated with Dalian University of Technology. The inclusion criteria were as follows: (i) patients aged 18 years or older and (ii) those undergoing dialysis for 3 or more months. The exclusion criteria were as follows: (i) patients with active systemic infection (n = 78), (ii) those with cardiovascular events in the last 3 months (n = 72), (iii) those with malignancies (n = 15), (iv) those refusing study participation (n = 47), and (v) those refusing study compliance (n = 16). Finally, 521 MHD patients were enrolled in this study (Fig. 1). All participants were receiving maintenance dialysis for 4 h three times per week. The blood flow rate was 200–300 mL/min, and the dialysate flow rate was 500 mL/min. This study was approved by the ethics committee of Dalian Municipal Central Hospital and all participants signed informed consent forms.

### Data collection

Baseline demographic and clinical information (age, sex, body mass index [BMI], primary cause of ESRD, comorbidities, and dialyzing mode [high-flux or low-flux dialyzation]) was collected. Blood was collected before dialysis during the midweek dialysis day to measure the levels of albumin (Alb), creatinine (Cr), blood urea nitrogen (BUN), and C-reactive protein (CRP) by standard laboratory procedures and hemoglobin (Hb) by the sodium dodecyl lauryl sulfate method. Serum β_2_M levels were measured by latex immunoassay. The adequacy of dialysis was calculated by measuring urea clearance (Kt/v) using a standard laboratory procedure [24].

### Outcome evaluation

Patients were followed up until December 2021. During the follow-up period, the primary outcome was all-cause and CVD mortality, whereas the secondary outcome was CVEs. CVD-related deaths included those from sudden cardiac death, heart failure, myocardial infarction, serious arrhythmias, and cerebrovascular accidents. CVEs were included incidence of coronary artery disease, congestive heart failure, arrhythmia, non-fatal cerebrovascular disease during follow-up in patients free of CVD at baseline. All deaths and events were accurately recorded using Therapy Support Suite 2.0 (Baden Humboldt, German), B-Soft Enterprise Application Portal 5.5 (Hangzhou, China), and Chinese National Renal Data System 2017 (Beijing, China). For each patient, the time to event was calculated as the time from the date of entry into the study to the date of death, the time of kidney transplantation, the date of quitting this study, or the end of this study, whichever came first.

### Statistical analysis

Serum β_2_M levels were categorized into three tertiles, and the lowest tertile served as the reference group. The normality of all continuous variables was evaluated using the Shapiro–Wilk statistic. The results of continuous variables were expressed as mean ± standard deviation (SD) or median [interquartile range (IQR)], and intergroup comparisons were analyzed by one-way ANOVA for normally distributed data or Kruskal–Wallis H test for non-normally distributed data. Categorical variables were expressed as counts with percentages, and differences between the two groups were examined using chi-square test.

Survival curves were calculated by the Kaplan–Meier method, and differences between the curves were analyzed using the log-rank test. We used the Schoenfeld residual test to verify the assumption of proportional hazards in the Cox analysis, and no violations were found (all *P* > 0.05). Multivariable Cox proportional hazard regression models were used to calculate the hazards ratios (HRs) and the corresponding 95% confidence intervals (CIs). The models were without any adjustment (crude); adjusted for age, gender and duration of dialysis (model 2); and additionally adjusted for Hb, Alb, Cr, CRP, BMI, primary disease, hypertension, diabetes, dialyzation mode on the basis of model 2 (model 3). After excluding CVD patients at baseline, we investigated the association between serum β_2_M levels and the incidence of CVEs. In addition, we performed sensitivity analysis by excluding CVD patients at baseline. Statistical significance was set at *P* < 0.05 and based on a two-sided test. All analyses were carried out using SAS 9.4 software (SAS Institute, Inc., Cary, NC, USA).

## Results

The duration of follow-up was 21.4 ± 6.3 months. During the follow-up period, there were 106 deaths, of which 68 were caused by cardiovascular disease. Of the 335 patients free of CVD at baseline, 66 had the incident CVEs. The baseline patient characteristics are shown in Table 1. The median (interquartile) serum β_2_M concentration was 40.1 (17.1) mg/L, the median (interquartile) patient age was 60 (19) years, and the median (interquartile) dialysis duration was 54 (67) months. Patients in the highest β_2_M tertile (> 45.4 mg/L) tended to be older, have longer dialysis durations and higher serum CRP levels, and require more low-flux dialyzation than patients in the reference group (< 34.9 mg/L). In addition, patients in the highest β_2_M tertile were more likely to have lower BMI, as well as lower serum Hb and Cr levels, and require less high-flux dialyzation.

**Table 1:** Baseline characteristics of the study patients according to serum β_2_M levels

Characteristic	Study population (n = 521)	β_2_M range (mg/L)			
		**T1 (< 34.9)** **(n = 173)**	**T2 (34.9–45.4)** **(n = 172)**	**T3 (> 45.4)** **(n = 176)**	***P***
Age, years	60 (19)	57 (20)	60 (19)	62 (17)	0.02
Male, n (%)	303 (58.1)	106 (61.3)	97 (56.4)	100 (56.8)	0.60
BMI, Kg/m^2^	23.4 (5.2)	24.3 (4.9)	23.6 (5.7)	22.5 (5.2)	< 0.01
Dialysis duration, months	54 (67)	46 (66)	53 (75)	62 (60)	< 0.01
Primary disease, n (%)					0.28
Diabetic nephropathy	167 (32.1)	57 (33.0)	54 (31.4)	56 (31.8)	
Glomerulonephritis	214 (41.1)	77 (44.5)	69 (40.1)	68 (38.6)	
Hypertensive benign renal arteriosclerosis	78 (15.0)	23 (13.3)	32 (18.6)	23 (13.1)	
Others	62 (11.7)	16 (9.2)	17 (9.9)	29 (16.5)	
hypertension status, n (%)	376 (72.2)	121 (69.4)	129 (75.0)	126 (71.6)	0.49
diabetes mellitus status, n (%)	178 (34.1)	64 (37.0)	62 (36.1)	52 (29.6)	0.56
dialyzer mode, n (%)					< 0.01
high-flux dialyzers	190 (36.5)	110 (63.6)	62 (36.1)	18 (10.2)	
low-flux dialyzers	331 (62.3)	63 (36.4)	110 (64.0)	158 (89.8)	
Hemoglobin, g/L	112 (19)	114 (15)	110 (20)	112 (22)	0.01
Albumin, g/L	41.8 (4.3)	42 (3.7)	41.7 (4.6)	41.9 (4.1)	0.66
Urea nitrogen, mmol/L	26.1 (7.6)	25.4 (6.6)	26.3 (7.5)	26.7 (9.1)	0.06
Creatinine, umol/L	932.3 ± 244.3	939.4 ± 262.8	927.8 ± 243.4	930.1 ± 227.0	< 0.01
CRP, ug/L	3.1 (5.2)	3.1 (2.1)	3.1 (6.8)	4.0 (6.6)	< 0.01
Kt/v	1.3 (0.3)	1.3 (0.3)	1.3 (0.3)	1.3 (0.4)	0.79
β_2_M, mg/L	40.1 (17.1)	29.7 (5.3)	40.1 (5.9)	53.1 (9.4)	< 0.01

Abbreviations: BMI, body mass index, CRP, C-reactive protein, β_2_M, β_2_-microglobulin, T, tertile

Note: Data are displayed as mean ± standard deviation or median [IQR] for continuous variables and number (percent) for categorical variables

P values were determined with ANOVA test or Kruskal-Wallis H tests for continuous variables and chi-square test for categorical variables

All statistical tests are two sided

**Fig. 2:**
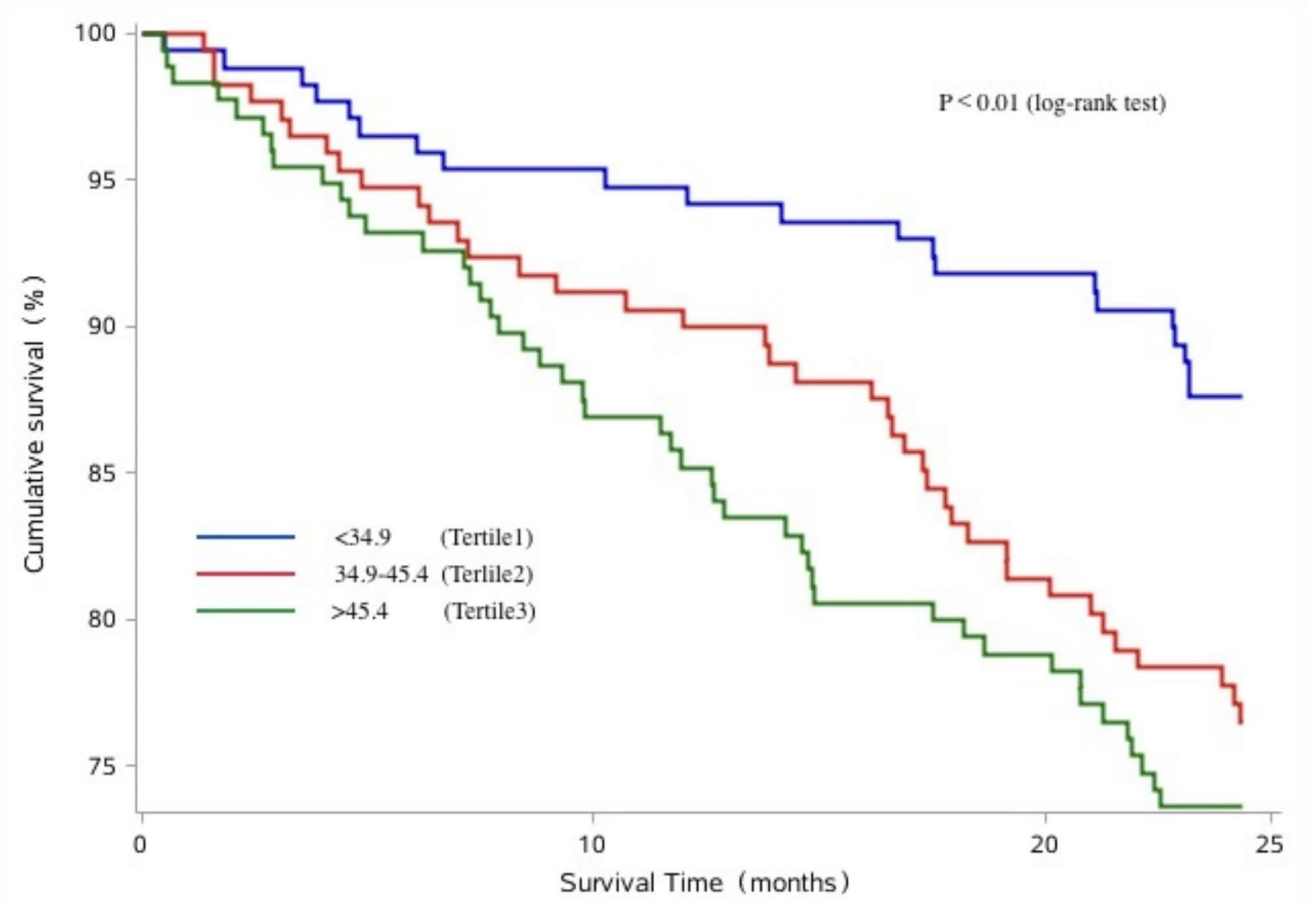
Kaplan–Meier analysis of all-cause mortality of 521 maintenance hemodialysis patients, classified according to tertiles of serum β_2_M levels

**Fig. 3:**
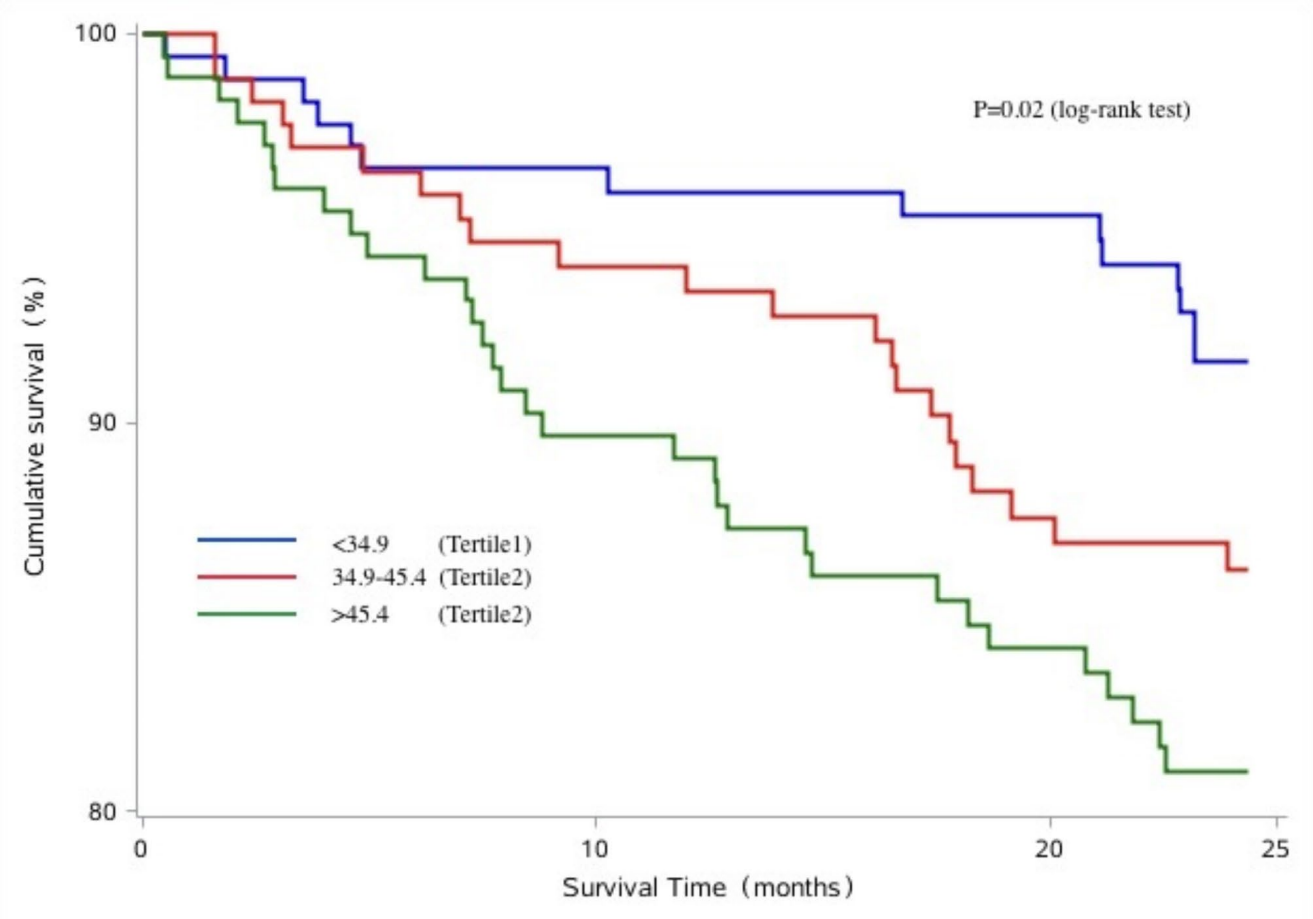
Kaplan–Meier analysis of cardiovascular disease mortality of 521 maintenance hemodialysis patients, classified according to tertiles of serum β_2_M levels

**Fig. 4:**
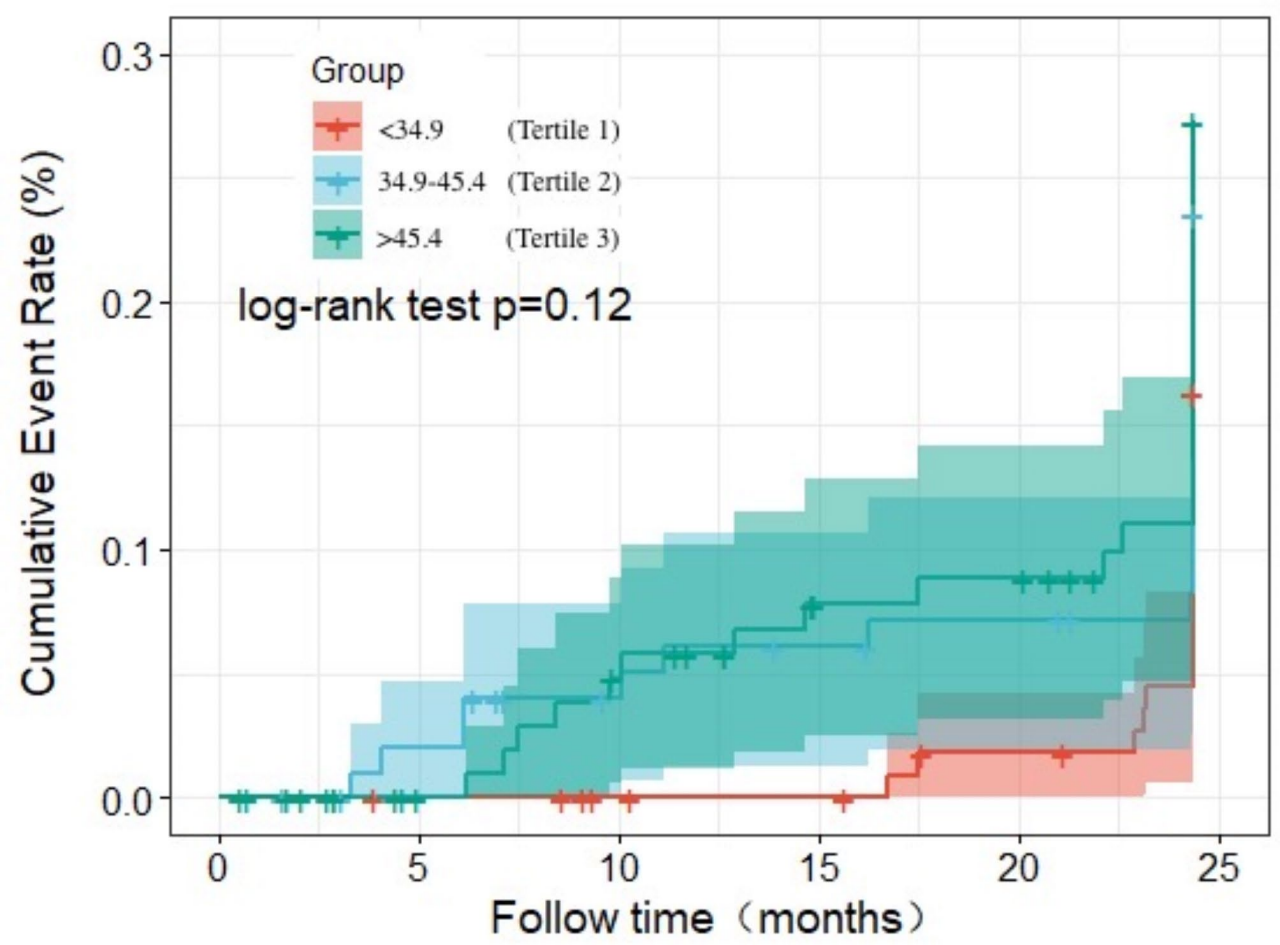
Kaplan–Meier analysis of cardiovascular events of 335 maintenance hemodialysis patients, classified according to tertiles of serum β_2_M levels

The Kaplan–Meier survival curves of patients with different serum β_2_M levels are shown in Figs. 2 and 3, and 4. A greater number of patients associated with the risk of all-cause and CVD mortality in the highest β_2_M tertile compared with the lowest β_2_M tertile (log-rank test, *P* < 0.05). However, we didn’t observed the significant association between serum β_2_M levels and CVEs (log-rank test, *P* > 0.05).

The associations of serum β_2_M levels with the risk of all-cause and CVD mortality are shown in Table 2. The highest β_2_M tertile showed an increase in all-cause mortality risk (HR = 2.24, 95% CI = 1.21–4.17) and CVD mortality risk (HR = 2.54, 95% CI = 1.19–5.43) compared with the reference group after adjusting for the aforementioned confounders, and a linear trend was also evident (*P* < 0.05).

**Table 2:** Hazard ratios (95% CIs) for the association between serum β_2_M levels and all-cause and cardiovascular disease mortality

Categories	Model 1^a^ HR(95% CI)	Model 2^b^ HR(95% CI)	Model 3^c^ HR(95% CI)
All-cause mortality			
T1	1.00 (Ref)	1.00 (Ref)	1.00 (Ref)
T2	2.01 (1.18–3.42)	1.88 (1.10–3.20)	1.95 (1.11–3.42)
T3	2.35 (1.40–3.94)	2.17 (1.29–3.68)	2.24 (1.21–4.17)
* P* for trend	< 0.01	< 0.01	0.02
Cardiovascular mortality			
T1	1.00 (Ref)	1.00 (Ref)	1.00 (Ref)
T2	1.69 (0.87–3.31)	1.66 (0.84–3.26)	1.73 (0.85–3.52)
T3	2.44 (1.30–4.57)	2.41 (1.28–4.57)	2.54 (1.19–5.43)
* P* for trend	< 0.01	< 0.01	0.02

Abbreviations: CI, confidence interval; HR, hazards ratio; Ref, reference; T, tertile, β_2_M, β_2_-microglobulin

^a^ Model 1: crude model

^b^ Model 2: adjusted for age, gender and dialysis duration

^c^ Model 3: adjusted for age, gender, dialysis duration, hemoglobin, albumin, creatinine, C-reactive protein, body mass index, primary disease, hypertension, diabetes, dialysis pattern

The association between serum β_2_M levels and CVEs is shown in Table 3. There were also trends towards higher incidence of CVEs in the highest β_2_M tertile compared with the reference group, although we did not observe a significant difference (HR = 1.62, 95% CI = 0.75–3.46).

**Table 3:** Hazard ratios (95% CIs) for the association between serum β_2_M levels and cardiovascular events in hemodialysis patients

Categories	Model 1^a^ HR(95% CI)	Model 2^b^ HR(95% CI)	Model 3^c^ HR(95% CI)
Cardiovascular Events			
T1	1.00 (Ref)	1.00 (Ref)	1.00 (Ref)
T2	1.55 (0.83–2.89)	1.39 (0.74–2.61)	1.52 (0.75–3.06)
T3	1.85 (1.01–3.37)	1.59 (0.86–2.95)	1.62 (0.75–3.46)
* P* for trend	0.05	0.15	0.25

Abbreviations: CI, confidence interval; HR, hazards ratio; Ref, reference, T, tertile, β_2_M, β_2_-microglobulin

^a^ Model 1: crude model

^b^ Model 2: adjusted for age, gender and dialysis duration

^c^ Model 3: adjusted for age, gender, dialysis duration, hemoglobin, albumin, creatinine, C-reactive protein, body mass index, primary disease, hypertension, diabetes, dialysis pattern

Sensitivity analysis of the associations of serum β_2_M levels with the risk of all-cause and CVD mortality are shown in Table 4. The results of sensitivity analysis were consistent with the main findings, indicating that our results are reliable.

**Table 4:** Hazard ratios (95% CIs) for the association between serum β_2_M levels and all-cause and cardiovascular disease mortality when excluding cardiovascular disease patients at baseline

Categories	Model 1^a^ HR(95% CI)	Model 2^b^ HR(95% CI)	Model 3^c^ HR(95% CI)
All-cause mortality			
T1	1.00 (Ref)	1.00 (Ref)	1.00 (Ref)
T2	1.78 (0.76–4.16)	1.49 (0.63–3.54)	1.49 (0.57–3.91)
T3	3.79 (1.80–8.01)	3.25 (1.52–6.96)	2.83 (1.10–7.26)
* P* for trend	< 0.01	< 0.01	< 0.02
Cardiovascular mortality			
T1	1.00 (Ref)	1.00 (Ref)	1.00 (Ref)
T2	1.63 (0.57–4.70)	1.57 (0.54–4.60)	1.33 (0.41–4.29)
T3	3.86 (1.55–9.63)	3.88 (1.54–9.79)	2.68 (0.88–8.13)
* P* for trend	< 0.01	< 0.01	< 0.04

Abbreviations: CI, confidence interval; HR, hazards ratio; Ref, reference, T, tertile, β_2_M, β_2_-microglobulin

^a^ Model 1: crude model

^b^ Model 2: adjusted for age, gender and dialysis duration

^c^ Model 3: adjusted for age, gender, dialysis duration, hemoglobin, albumin, creatinine, C-reactive protein, body mass index, primary disease, hypertension, diabetes, dialysis pattern

## Discussion

In the current observational study, we examined the associations between serum β_2_M levels and the risk of all-cause and CVD mortality and the incidence of CVEs among MHD patients. The findings indicated that higher serum β_2_M levels were independently associated with higher risk of all-cause and CVD mortality, but not for the incidence of CVEs after adjusting for potential confounders.

Several studies have explored the associations between serum β_2_M levels and the risk of all-cause and CVD mortality among MHD patients; however, the results were inconclusive. In the DOPPS study, Kanda et al. recruited 5332 MHD patients, of which 15.4% required hemodiafiltration, and demonstrated that the highest serum β_2_M levels (> 29 mg/L) were associated with higher mortality compared with those with the lowest serum β_2_M levels (≤ 23 mg/L), consistent with our results [25]. By contrast, Kim et al. conducted a retrospective cohort study of 289 MHD patients in Korea, of which 94% required low-flux dialyzation, and the mean serum β_2_M level was (35.4 ± 12.7) mg/L, indicating that elevated serum β_2_M levels decreased the risk of all-cause mortality, which was contrary to our results [21]. The difference in results between studies may be due to the sample size, that is, the number of patients in their study was smaller than that in our study. In terms of the relationship between serum β_2_M levels and CVD mortality, several studies have reported that serum β_2_M levels were not associated with CVD mortality, which was inconsistent with our study. For instance, Okuno et al. conducted a prospective cohort study of 490 MHD patients with a mean serum β_2_M level of (32.5 ± 7.2) mg/L and indicated that a higher serum β_2_M level was not a predictor of a higher risk of mortality from CVD in Japan [26]. The inconsistency with our study may be due to the different modes of dialysis that the patients were undergoing. It is difficult to clear β_2_M with low-flux dialyzation [13]. The patients in Japan were treated with high-flux dialyzers, whereas patients in our study were treated with both high-flux and low-flux dialyzers. Furthermore, Cheung et al. conducted a prospective cohort study of 1813 MHD patients with a mean serum β_2_M level of (37.6 ± 11.9) mg/L in the United States, and they also indicated that there was no association between the serum β_2_M level and the risk of mortality from CVD in both low-flux (50%) and high-flux (50%) groups [27]. This difference may be due to the differences in race and dialysis duration. The dialysis duration of patients in our study was longer (5.8 ± 4.1 years) than that in their study (3.8 ± 4.4 years). Serum β_2_M levels may also increase with the increase in dialysis duration [28]. As for the relationship between serum β_2_M levels and CVEs, a cohort study in the SPRINT trial recruited 2377 CKD patients, and found that serum β_2_M levels were not associated with the rate of CVEs [23], which was consistent with our results. However, in the CRIC study, Foster et al. recruited 3613 CKD patients and indicated that higher serum β_2_M levels was associated with increased incidence of CVEs [22], which was contrary to our results. This might be due to the difference of study population, race, and sample size.

The significance of β_2_M in mortality has been unclear until recently. According to previous studies, β_2_M damages vessels by facilitating amyloid deposition within vessel walls [29]. In patients with peripheral arterial disease, serum β_2_M levels are elevated, correlating with the severity of disease independent of other risk factors. In addition, β_2_M induces the formation of glycosylated end products, which are substrates for oxidative injury, thereby further contributing to the proatherogenic milieu of uremia [30]. Serum β_2_M levels are positively correlated with carotid atherosclerosis severity in MHD patients [31]. Moreover, the uremic milieu has a negative impact on the vasculatory system. Serum β_2_M levels were inversely correlated with the number of CD34^+^ CD133^+^ immature progenitor cells in MHD patients, and these cells contributed to vessel repair and neovascularization [32]. Furthermore, the uremic milieu may disrupt vascular repair in patients with kidney failure [33]. β_2_M is also an initiator of inflammatory responses that can trigger inflammatory processes [34]. For instance, β_2_M stimulates monocytes to secrete high levels of proinflammatory cytokines such as tumor necrosis factor-α and interleukins-1, -6, -8, and − 10 [35]. High serum β_2_M levels induce apoptosis or necrosis in normal cells, including endothelial cells and fibroblasts [36]. Therefore, β_2_M may have direct harmful effects in MHD patients.

Our study had several strengths. First, this study is the first to investigate the role of serum β_2_M levels in predicting the risk of all-cause and CVD mortality and the incidence of CVEs in MHD patients in China. Second, our study was a prospective study conducted in a well characterized cohort of MHD patients from which detailed demographic and clinical information on comorbidities and laboratory indicators were collected, which allowed us to explore the associations between serum β_2_M levels and the risk of all-cause and CVD mortality and the incidence of CVEs with greater accuracy. Third, reasonable statistical techniques were applied, including sensitivity analysis to further validate our results. However, there were also some limitations in our study. First, serum β_2_M levels were measured at a single time point, which may be not reflect substantial intra-individual variability over time. But the findings can provide some clues for future studies. Second, due to the limited data, we failed to collect data on NT-proBNP and information on residual renal function, smoking history, and alcohol consumption. However, compared to previous studies, these shortcomings seemed to have little impact on our results [20, 26]. As with all observational studies, in spite of the many important confounding factors that were adjusted, we could not exclude potential confounders and residual confounders. Third, the enrolled patients were all from a single center, and the results may not be extrapolated into the overall MHD population. Finally, our study is an observational study, we were unable to establish the causality of the relationship between serum β_2_M levels and clinical outcomes, and the follow-up was short. Therefore, future studies are needed to confirm our findings.

## Conclusions

In conclusion, our study demonstrated that high serum β_2_M levels were a significant predictor of the risk of all-cause and CVD mortality in MHD patients, but not for the incidence of CVEs, indicating the clinical importance of lowering serum β_2_M in MHD patients. Increased efforts, particularly detailed prevention strategies to reduce serum β_2_M levels, should be applied to reduce the risk of all-cause and CVD mortality in the future.

## Data Availability

The datasets used and/or analyzed during the current study are available from the corresponding author upon reasonable request.

## Abbreviations

Alb: albumin
BMI: body mass index
BUN: blood urea nitrogen
CIs: confidence intervals
CKD: chronic kidney disease
Cr: creatinine
CRP: C-reactive protein
CVD: cardiovascular disease
ESRD: End stage renal disease
GFR: glomerular filtration rate
Hb: hemoglobin
HRs: Hazard ratios
IQR: interquartile range
Kt/v: urea clearance
MHD: maintenance hemodialysis
SD: standard deviation
UTs: uremic toxins
uremic toxins; β_2_M: β_2_-microglobulin
